# Supplementary material for: Deleterious Mutations Accumulate Faster in Allopolyploid Than Diploid Cotton (Gossypium) and Unequally between Subgenomes
Source: Mol Biol Evol. 2022 Jan 31;39(2):msac024. doi: 10.1093/molbev/msac024 (PMC8841602; doi:10.1093/molbev/msac024)
Supplement: msac024_Supplementary_Data [file msac024_supplementary_data.zip › Conover_Wendel_Supplemental_Files.pdf]

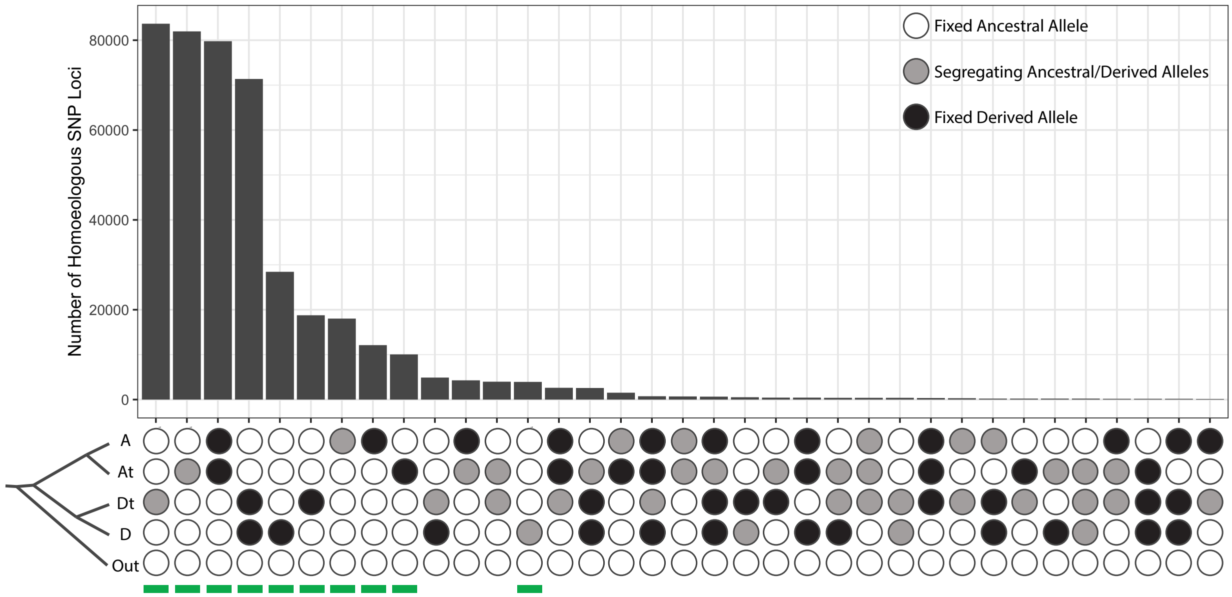

Supplementary Figure 1: UpSet Plot of Derived Homoeologous SNPs Among 8,884 Syntenic Homoeologous Gene Pairs

To identify SNPs that may have potentially arisen from causes other than simple nucleotide substitutions (e.g., sequencing error, gene conversion), we plotted the frequency of polarized (ancestral vs derived) SNPs across the four major clades of *Gossypium* allopolyploid genomes (A diploid, At subgenome, Dt subgenome, D diploid). Bottom of the UpSet plot shows the phylogenetic positions of these 4 groups, as well as the ancestral state used for polarization. For simplicity, we collapsed all polyploids into a single group, but split them by subgenome (e.g. the At row indicates the At subgenome in all 6 allopolyploids in this analysis). White bubbles indicate that only ancestral alleles were identified in that species or subgenome; black bubbles denoteSNP sites where only derived alleles were identified; grey bubbles represent SNP sites where both ancestral and derived alleles were identified. Only the top 35 SNP groups are shown. Groups with a green line underneath indicate SNP patterns that can be explained by a single mutational event with no homoplasy (e.g. from incomplete lineage sorting or recurrent mutation), and were retained for subsequent analyses involving the 8,884 homoeologous gene pairs.

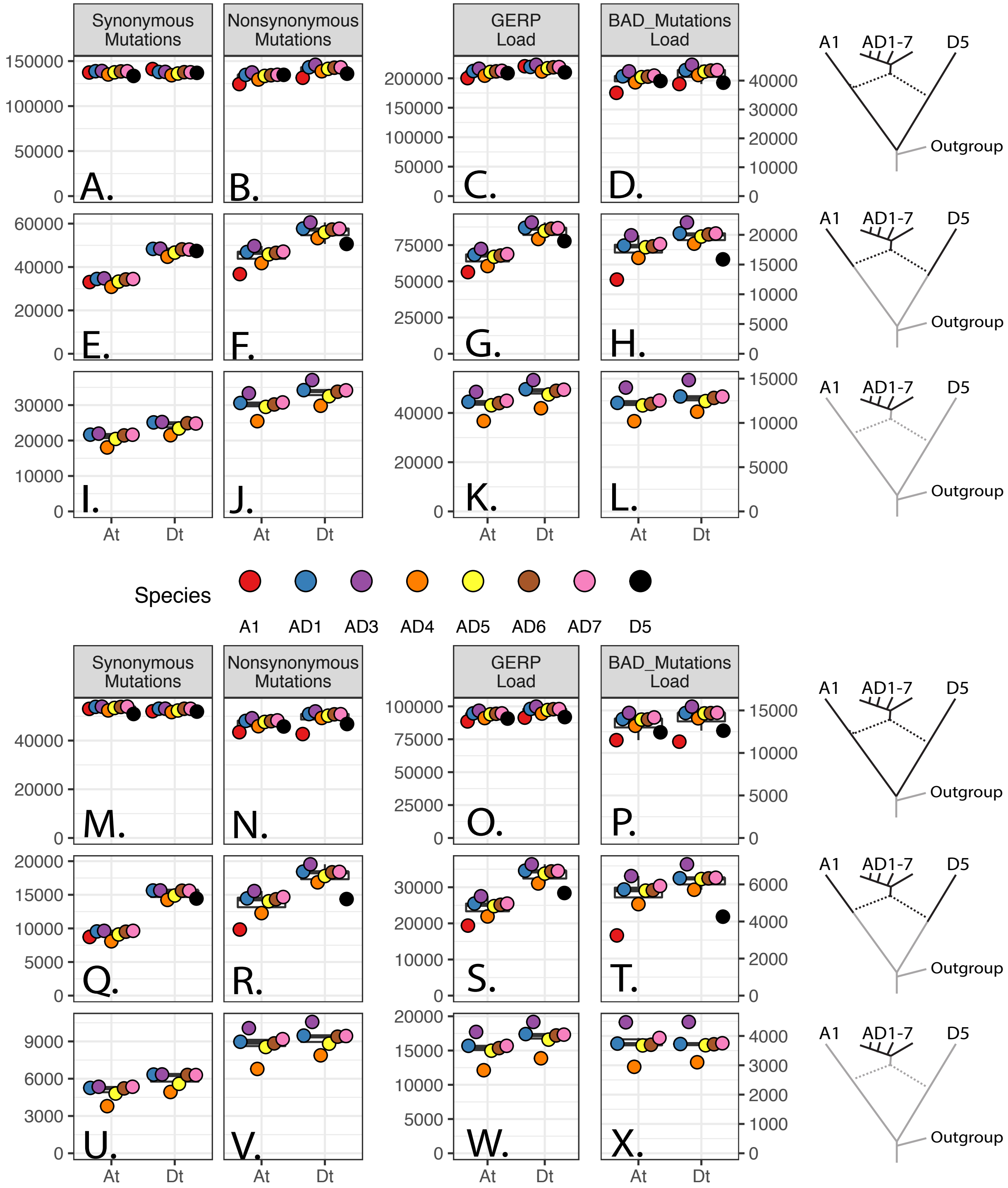

Supplementary Figure 2: Genome-Wide Derived Mutations and Deleterious Loads at Three Phylogenetic Depths

Number of derived synonymous, nonsynonymous, and deleterious mutations in the CDS regions of 8,884 pairs of homoeologs (17,768 genes in total) in eight cotton species at three phylogenetic depths (indicated by bold branches of phylogeny at right). For all panels, the ancestral state of each SNP was determined using three Australian

cottons as an outgroup (see Methods). The deepest phylogenetic depth **(ABCD)** includes all derived mutations that originated since the divergence of the A and D diploid progenitors; the middle row **(EFGH)** shows SNPs that are variable within each subgenome and its associated progenitor diploid species; and the bottom row **(IJKL)** shows SNPs that originated post-polyploidy and are variable within the polyploids. **(AEI)** Synonymous mutations. **(BFJ)** Nonsynonymous mutations. The y-axis for both synonymous and nonsynonymous is shown at left, and represents the sum of the derived allele frequencies, interpreted as the average number of derived SNPs in that category in each species. **(CGK)** GERP Load of each species, calculated as the sum of (derived allele frequency * GERP Score) for all SNP positions with GERP > 0. **(DHL)** Number of deleterious mutations in each species, calculated by BAD_Mutations with bonferroni corrected significance (see Methods). Y-axis represents the sum of the derived allele frequencies, and indicates the average number of deleterious mutations in each species at a given phylogenetic depth. **Note:** for **(EFGH)**, comparisons between subgenomes cannot be made because the D5 diploid is more distantly related to the D subgenome than the A1 diploid is related to the A subgenome. Therefore, we would expect a larger number of derived mutations in D than A simply due to evolutionary history rather than to polyploidization *per se*. The panels above the figure legend are identical to those presented in Figure 2. The panels below the figure legend **(M-X)** follow the same order as **(A-L)**, but represent the genome-wide totals without any filtering based on homoeologs or potential sites that are due to gene less, mapping biases, or homoeologous gene conversion and is provided to demonstrate that our filtering criteria did not have a noticeable impact on the patterns of SNPs that we observed, and that homoeologous interactions have a minimal effect on patterns of evolution following allopolyploidy in *Gossypium*.

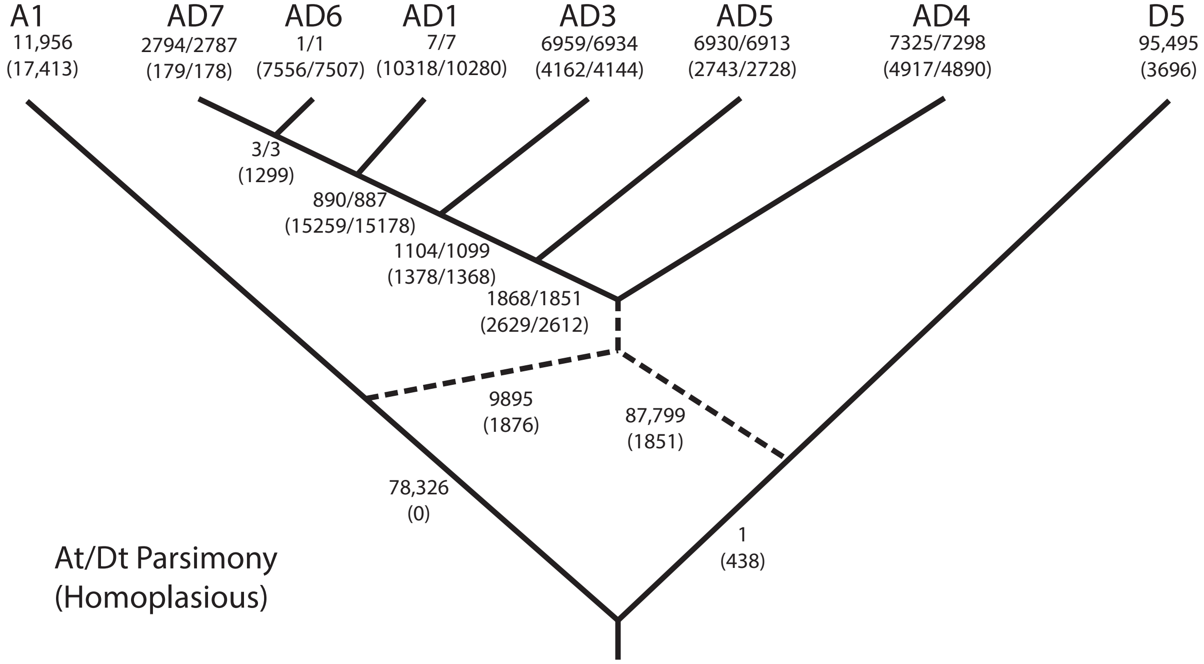

Supplementary Figure 3: Phylogenetic Positions of Derived Deleterious SNPs
For SNPs that passed the filtering from Supplementary Figure 1, we placed the origin of the SNP on the phylogenetic tree using parsimony. Numbers in the format of “X/Y” indicate the number of SNPs found in the “At/Dt” subgenome. Numbers above the parentheses indicate SNPs that are unequivocally placed on the tree in either the At or Dt subgenome. Numbers in parentheses indicate SNPs that are homoplasious, and the position of the number represents the phylogenetic position of the most recent common ancestor of all species that contain at least one derived SNP. Numbers in the parentheses at the tips of the tree indicate SNPs that are segregating within that species but are not found in any other species. Note: the high amount of homoplasious SNPs at the base of the AD1, AD6, and AD7 clade is most likely caused by recent hybridization or introgression of AD1 into AD6, as also indicated in Supplementary Figure 5.

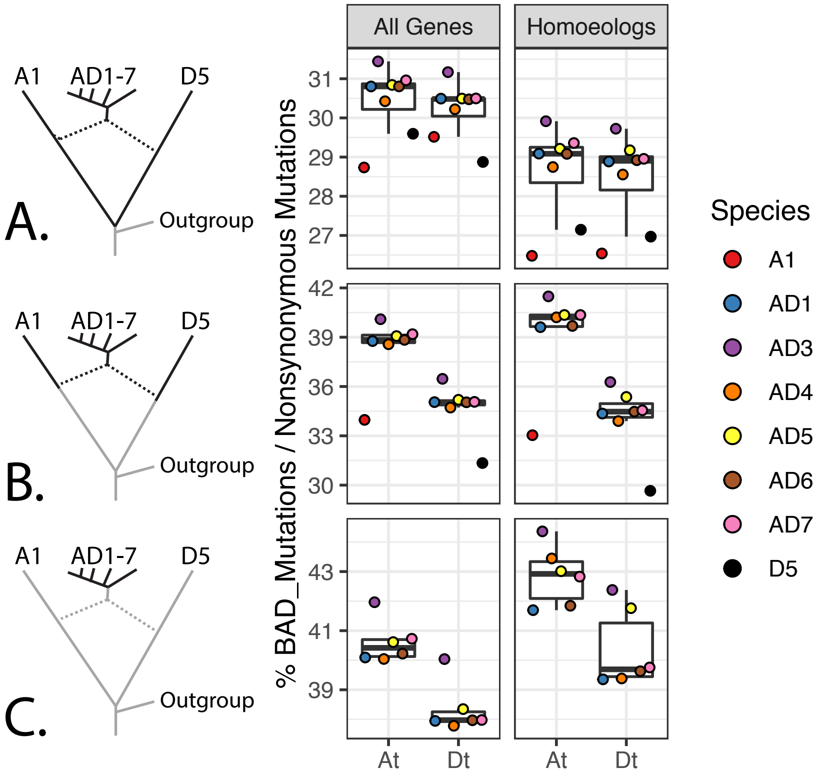

Supplementary Figure 4: Genome-Wide Proportions of All Nonsynonymous Mutations That Are Deleterious

Rows **A**, **B**, and **C** summarize SNPs segregating within the entire clade, within each subgenome and its respective progenitor diploid, and within each subgenome, as indicated by the bolded branches along the phylogeny at left. **(A)** Proportion of all nonsynonymous SNPs that are deleterious genome-wide within each subgenome. **(B)** Proportion of nonsynonymous SNPs that are deleterious within 8,884 homoeologous pairs (17,768 total genes) that are syntenically conserved between the two subgenomes of *G. hirsutum* (see Methods for filtering criteria). Note: Similar to Figure 2, comparisons between subgenomes in row **B** reflect differing phylogenetic distances, not asymmetries between the subgenomes and/or their diploid progenitors.

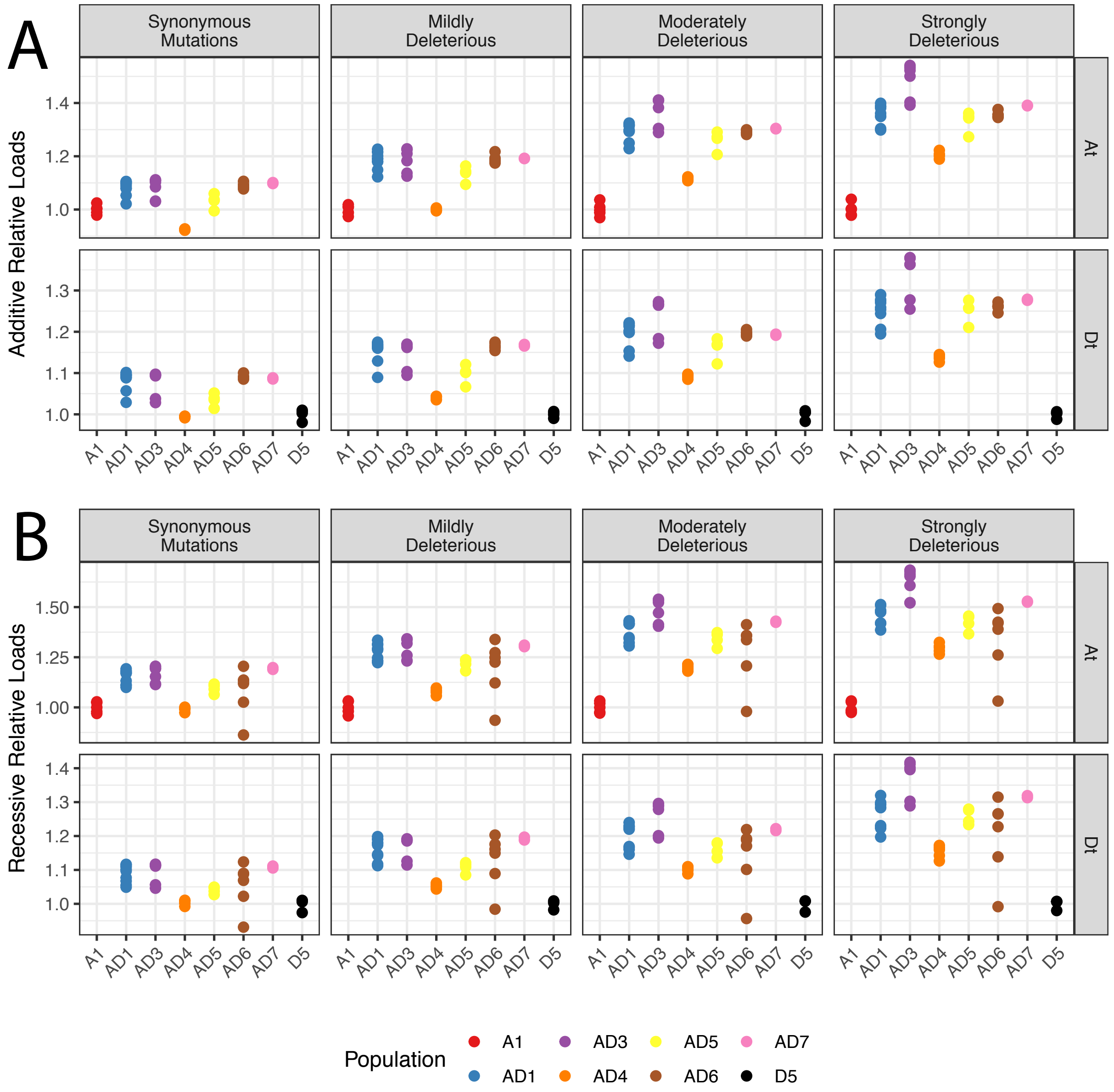

Supplementary Figure 5: Additive and Recessive Models of Deleterious Mutation Accumulation

Relative load of synonymous sites and varying GERP categories from an **(A)** additive model (i.e. counting all SNPs) and **(B)** recessive model (i.e. counting all homozygous SNPs in a homozygous state). Each point represents an individual, and the placement of each point represents the relative increase or decrease in the number of SNPs relative to the average of the number of SNPs in the diploid (A1 for At, D5 for Dt). Note: The high variance in the recessive load for AD6 reflects a high number of sites that are heterozygous. This is mostly likely due to recent hybridization or introgression from AD1, which is also indicated by a high amount of incomplete lineage sorting between AD1, AD6, and AD7 in Supplementary Figure 3.

Supplementary Table 1: of Accessions Used in This Study and SRA Codes

NCBI_ID	SampleID	Species	Clultivar Name
SRX3412081	BPS1152	*G. hirsutum (AD1)*	7200 BPS 1152
SRX3412083	BPS1173	*G. hirsutum (AD1)*	7221 BPS 1173
SRX3411942	BPS1244	*G. hirsutum (AD1)*	7292 BPS 1244
SRX3411841	TX0967	*G. hirsutum (AD1)*	TX-0967
SRX3411844	TX0969	*G. hirsutum (AD1)*	TX-0969
SRX3411860	TX1037	*G. hirsutum (AD1)*	TX-1037
SRX3412068	TX1996	*G. hirsutum (AD1)*	TX-1996
SRX3411953	TX2091	*G. hirsutum (AD1)*	TX-2091
SRX3411951	TX2093	*G. hirsutum (AD1)*	TX-2093
SAMN04998813	TX2094	*G. hirsutum (AD1)*	G. h. yucatanense TX2094
SRX3433856	AD3-14	*G. tomentosum (AD3)*	AD3-14
SRX3433661	AD3-25	*G. tomentosum (AD3)*	AD3-25
SRX3433674	AD3-26	*G. tomentosum (AD3)*	AD3-26
SRX3433648	AD3-23	*G. tomentosum (AD3)*	AD3-23
SRX3433678	AD3-16	*G. tomentosum (AD3)*	AD3-16
SRX3433647	AD3-07	*G. tomentosum (AD3)*	AD3-07
SRX996777	AD3	*G. tomentosum (AD3)*	*G. tomentosum*
SRX3433649	AD4-10	*G. mustelinum (AD4)*	AD4-10
SRX3433721	AD4-W	*G. mustelinum (AD4)*	AD4-W
SRX3433650	AD4-16	*G. mustelinum (AD4)*	AD4-16
SRX996778	AD4	*G. mustelinum (AD4)*	*G. mustelinum*
SAMN04998799	AD4-8	*G. mustelinum (AD4)*	*G. mustelinum* AD4-8
SRX3433804	AD5-2	*G. darwinii (AD5)*	AD5-2

SRX3433805	AD5-CB	*G. darwinii (AD5)*	AD5-CB
SRX996779	AD5	*G. darwinii (AD5)*	*G. darwinii*
SRS705696	G. darwinii	*G. darwinii (AD5)*	*G. darwinii*
SRX3433726	TX2265	*G. ekmanianum (AD6)*	TX2265
SRX3433727	TX2266	*G. ekmanianum (AD6)*	TX2266
SRX3433722	TX2271	*G. ekmanianum (AD6)*	TX2271
SRX996780	TX2263	*G. ekmanianum (AD6)*	*G. ekmanianum* (TX-2263)
SAMN04998801	AD6	*G. ekmanianum (AD6)*	*G. ekmanianum*
SRX3433724	TX2273	*G. ekmanianum (AD6)*	TX2273
SRX996781	AD7	*G. stephensii (AD7)*	Wake Island Atoll Accession
SRX996782	P1B	*G. stephensii (AD7)*	Pearl1B
SRR847961	D5_2	*G. raimondii (D5)*	D5_2
SRR847980	D5_31	*G. raimondii (D5)*	D5_31
SRR847974	D5_4	*G. raimondii (D5)*	D5_4
SRR847981	D5_53	*G. raimondii (D5)*	D5_53
SRR8979982	A1_073	*G. herbaceum (A1)*	A1_073
SRR8979983	A1_074	*G. herbaceum (A1)*	A1_074
SRR8979969	A1_155	*G. herbaceum (A1)*	A1_155
SRR8979967	A1_Af	*G. herbaceum (A1)*	A1_Af
SRR8979966	A1_Nisa	*G. herbaceum (A1)*	A1_Nisa
SRR8979901	C2rob_ISU1024	*G. robinsonii (C2)*	C2_4
SRR8979997	G2aus_BW21	*G. australe (G2)*	G2-21
SRR8979903	ISU1019	*G. nelsonii (G3)*	G3-83

Supplementary Table 2: Species Used in GERP Score Calculation

Species used	Link to Genome Sequence
Gossypium hirsutum v1.1	http://genome.jgi.doe.gov/pages/dynamicOrganismDownload.jsf?organism=Ghirsutum
Gossypium raimondii v2.1	http://genome.jgi.doe.gov/pages/dynamicOrganismDownload.jsf?organism=Graimondii
Vitis vinifera 12X	http://genome.jgi.doe.gov/pages/dynamicOrganismDownload.jsf?organism=Vvinifera
Fragaria vesca v1.1	http://genome.jgi.doe.gov/pages/dynamicOrganismDownload.jsf?organism=Fvesca
Citrus maxima	ftp://ftp.ncbi.nlm.nih.gov/genomes/all/GCA/002/006/925/GCA_002006925.1_ASM200692v1/GCA_002006925.1_ASM200692v1_genomic.fna.gz
Citrus sinensis v1.1	http://genome.jgi.doe.gov/pages/dynamicOrganismDownload.jsf?organism=Csinensis
Citrus clementina v1.0	http://genome.jgi.doe.gov/pages/dynamicOrganismDownload.jsf?organism=Cclementina
Cucumbis sativus	http://genome.jgi.doe.gov/pages/dynamicOrganismDownload.jsf?organism=Csativus
Theobroma cacao	http://genome.jgi.doe.gov/pages/dynamicOrganismDownload.jsf?organism=Tcacao
Prunus persica v2.1	http://genome.jgi.doe.gov/pages/dynamicOrganismDownload.jsf?organism=Ppersica
Carica papaya	http://genome.jgi.doe.gov/pages/dynamicOrganismDownload.jsf?organism=Cpapaya

Supplementary Table 3: Species Used in BAD_Mutations Pipeline

Species	Common Name	Assembly Version	Annotation Version	Source
Aegilops tauschii	Goatgrass	ASM34733v1	1	Ensembl Plants
Aquilegia coerulea	Columbine	1.1	1.1	Phytozome 10
Arabidopsis lyrata	Lyrate rockcress	1	1	Phytozome 10
Arabidopsis thaliana	Thale cress	TAIR10	TAIR10	Phytozome 10
Boechera stricta	Drummond's rockcress	1.2	1.2	Phytozome 10
Brachypodium distachyon	Purple false brome	2.1	2.1	Phytozome 10
Brassica oleracea	Cabbage	2.1	2.1	Ensembl Plants
Brassica rapa	Turnip mustard	FPsc 1.3	1	Phytozome 10
Capsella grandiflora	--	1.1	1.1	Phytozome 10
Capsella rubella	Red shepherd's purse	1	1	Phytozome 10
Carica papaya	Papaya	ASGPBv0.4	ASGPBv0.4	Phytozome 10
Citrus clementina	Clementine	1	clementine1.0	Phytozome 10
Citrus sinensis	Sweet orange	1	orange1.1	Phytozome 10
Cucumis sativus	Cucumber	1	1	Phytozome 10
Eucalyptus grandis	Eucalyptus	2	2	Phytozome 10
Eutrema salsugineum	Salt cress	1	1	Phytozome 10
Fragaria vesca	Strawberry	1.1	1.1	Phytozome 10
Glycine max	Soybean	a2	a2.v1	Phytozome 10

Hordeum vulgare	Barley	082214v1	1	Ensembl Plants
Leersia perrieri	Cutgrass	1.4	1	Ensembl Plants
Linum usitatissimum	Flax	1	1	Phytozome 10
Malus domestica	Apple	1	1	Phytozome 10
Manihot esculenta	Cassava	6	6.1	Phytozome 10
Medicago truncatula	Barrel medic	Mt4.0	Mt4.0v1	Phytozome 10
Mimulus guttatus	Monkey flower	2	2	Phytozome 10
Musa acuminata	Banana	MA1	MA1	Ensembl Plants
Oryza sativa	Asian rice	IRGSP-1.0	7	Phytozome 10
Panicum virgatum	Switchgrass	1	1.1	Phytozome 10
Phaseolus vulgaris	Common bean	1	1	Phytozome 10
Populus trichocarpa	Western poplar	3	3	Phytozome 10
Prunus persica	Peach	2	2.1	Phytozome 10
Ricinus communis	Castor bean	0.1	0.1	Phytozome 10
Setaria italica	Foxtail millet	2	2.1	Phytozome 10
Solanum lycopersicum	Tomato	SL2.50	iTAG2.3	Phytozome 10
Solanum tuberosum	Potato	3_2.1.10	3.4	Phytozome 10
Sorghum bicolor	Milo	2	2.1	Phytozome 10
Theobroma cacao	Cacao	1	1	Phytozome 10
Triticum urartu	Red wild einkorn	ASM34745v1	1	Ensembl Plants
Vitis vinifera	Grape	Genoscope.12X	Genoscope.12X	Phytozome 10
Zea mays	Maize	6a	6a	Phytozome 10
