# Supplementary material for: Deleterious Mutations Accumulate Faster in Allopolyploid Than Diploid Cotton (Gossypium) and Unequally between Subgenomes
Source: Mol Biol Evol. 2022 Jan 31;39(2):msac024. doi: 10.1093/molbev/msac024 (PMC8841602; doi:10.1093/molbev/msac024)
Supplement: msac024_Supplementary_Data [file msac024_supplementary_data.zip › Supplemental_Material.docx]

**Supplementary Figure 1: UpSet Plot of Derived Homoeologous SNPs Among 8,884 Syntenic Homoeologous Gene Pairs**

To identify mutations that may have potentially arisen from causes other than simple nucleotide substitutions (e.g., sequencing error, gene conversion), we plotted the frequency of polarized (ancestral vs derived) mutations across the four major clades of *Gossypium* allopolyploid genomes (A diploid, At subgenome, Dt subgenome, D diploid). Bottom of the UpSet plot shows the phylogenetic positions of these 4 groups, as well as the ancestral state used for polarization. For simplicity, we collapsed all polyploids into a single group, but split them by subgenome (e.g. the At row indicates the At subgenome in all 6 allopolyploids in this analysis). White bubbles indicate that only ancestral alleles were identified in that species or subgenome; black bubbles denote variant sites where only derived alleles were identified; grey bubbles represent variant sites where both ancestral and derived alleles were identified. Only the top 35 variant groups are shown. Groups with a green line underneath indicate mutation patterns that can be explained by a single mutational event with no homoplasy (e.g. from incomplete lineage sorting or recurrent mutation), and were retained for subsequent analyses involving the 8,884 homoeologous gene pairs. Groups with a yellow line underneath indicate possible candidates for gene conversion, among other explanations (incomplete lineage sorting, recurrent mutation, etc.) and were removed from further analysis.

**Supplementary Figure 2: Genome-Wide Derived Mutations and Deleterious Loads at Three Phylogenetic Depths Are Not Affected By Variant Filtering**

**Panels A-L are a reprint of Figure 2 in the manuscript. Panels M-X show the same data, in the same order as panels A-L, but represent the genome-wide totals without any filtering based on homoeologs or potential sites that are due to gene less, mapping biases, or homoeologous gene conversion and is provided to demonstrate that our filtering criteria did not have a noticeable impact on the patterns of mutations that we observed, and that homoeologous interactions have a minimal effect on patterns of evolution following allopolyploidy in Gossypium.**

**Supplementary Figure 3: Phylogenetic Positions of Derived Deleterious Mutations**

For mutations that passed the filtering from Supplementary Figure 1, we placed the origin of the mutation on the phylogenetic tree using parsimony. Numbers in the format of “X/Y” indicate the number of mutations found in the “At/Dt” subgenome. Numbers above the parentheses indicate mutations that are unequivocally placed on the tree in either the At or Dt subgenome. Numbers in parentheses indicate mutations that are homoplasious, and the position of the number represents the phylogenetic position of the most recent common ancestor of all species that contain at least one derived mutation. Numbers in the parentheses at the tips of the tree indicate mutations that are segregating within that species but are not found in any other species. Note: the high amount of homoplasious mutations at the base of the AD1, AD6, and AD7 clade is most likely caused by recent hybridization or introgression of AD1 into AD6, as also indicated in Supplementary Figure 5.


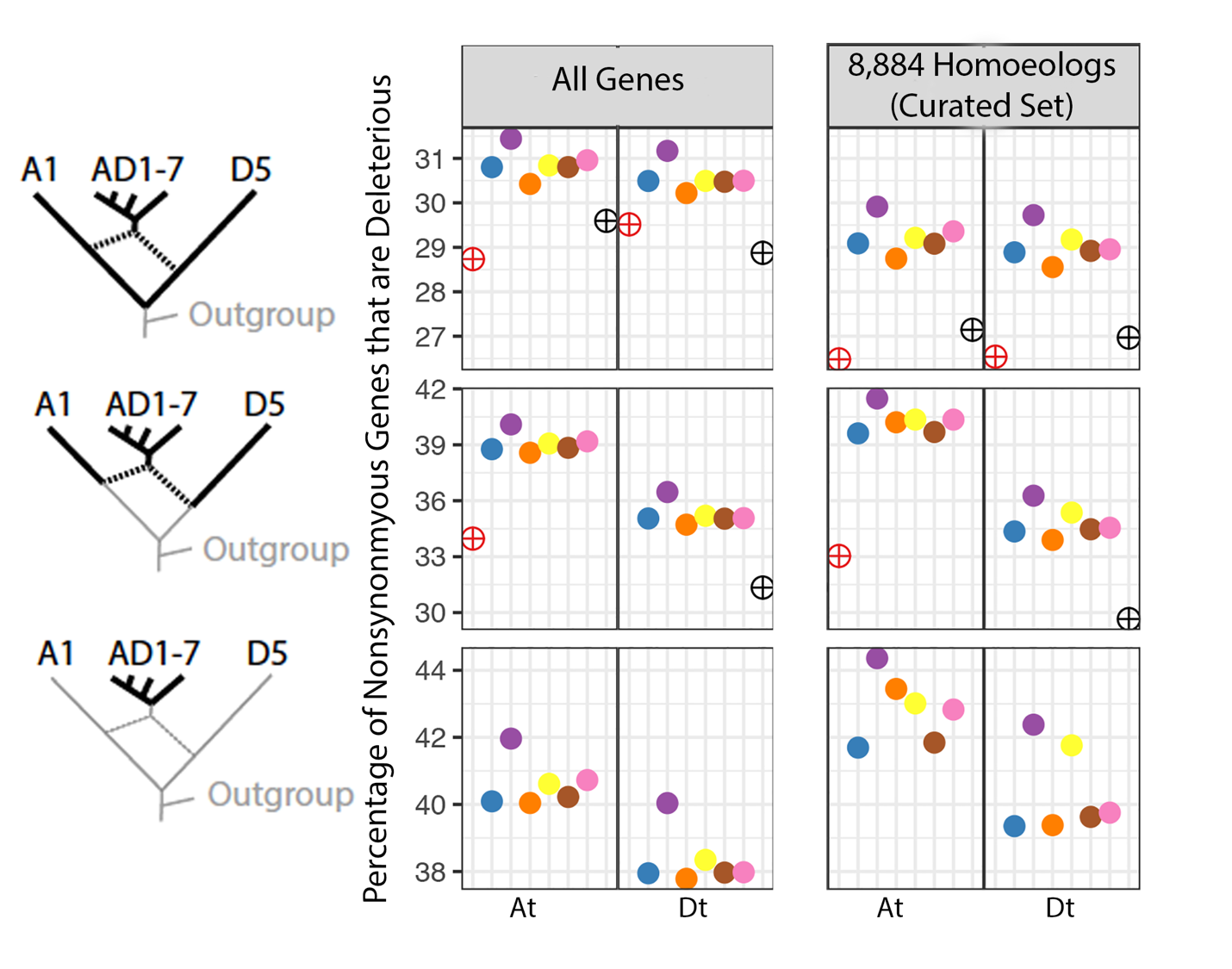


**Supplementary Figure 4: Genome-Wide Proportions of All Nonsynonymous Mutations That Are Deleterious**

Rows **A**, **B**, and **C** summarize mutations segregating within the entire clade, within each subgenome and its respective progenitor diploid, and within each subgenome, as indicated by the bolded branches along the phylogeny at left. **(A)** Proportion of all nonsynonymous mutations that are deleterious genome-wide within each subgenome. **(B)** Proportion of nonsynonymous mutations that are deleterious within 8,884 homoeologous pairs (17,768 total genes) that are syntenically conserved between the two subgenomes of *G. hirsutum* (see Methods for filtering criteria). Note: Similar to Figure 2, comparisons between subgenomes in row **B** reflect differing phylogenetic distances, not asymmetries between the subgenomes and/or their diploid progenitors.

**Supplementary Figure 5: Additive and Recessive Models of Deleterious Mutation Accumulation**

Relative load of synonymous sites and varying GERP categories from an **(A)** additive model (i.e. counting all mutations) and **(B)** recessive model (i.e. counting all mutations in a homozygous state). Each point represents an individual, and the placement of each point represents the relative increase or decrease in the number of mutations relative to the average of the number of mutations in the diploid (A1 for At, D5 for Dt). Note: The high variance in the recessive load for AD6 reflects a high number of sites that are heterozygous. This is mostly likely due to recent hybridization or introgression from AD1, which is also indicated by a high amount of incomplete lineage sorting between AD1, AD6, and AD7 in Supplementary Figure 3.

**Supplementary Figure 6**: Relative Increase Of Deleterious Mutations Among BAD_Mutations Constraint Values in Polyploids Compared to Diploids

For mutations that originated since the divergence of each subgenome from its diploid progenitor, we plotted the relative increase in deleterious alleles across values of “masked constraint” (i.e. codon-specific dN/dS values). Similar to Figure 4, we used the diploid as the reference population, meaning that the relative increase of the number of deleterious mutations in the diploid is always equal to one for all categories. In both subgenomes of all polyploids, mutations occurring within codons with lower dN/dS values had the greatest relative increase compared to the diploids, decreasing as the dN/dS values increased.

**Supplementary Table 1: List of Accessions Used in This Study and SRA Codes**

**Supplementary** [**Table**](https://docs.google.com/spreadsheets/u/0/d/1tq-eTkUust0zQ4K0ZqMrfWno7PbSTYgY2B-FMFYNYss/edit) **2: Species Used in GERP Score Calculation**

**Supplementary Table 3: Species Used in BAD_Mutations Pipeline**
